# Choi criteria are superior in evaluating tumor response in patients treated with transarterial radioembolization for hepatocellular carcinoma

**DOI:** 10.3892/ol.2013.1612

**Published:** 2013-10-10

**Authors:** ZHIHONG WENG, JUDITH ERTLE, SHAOPING ZHENG, THOMAS LAUENSTEIN, STEFAN MUELLER, ANDREAS BOCKISCH, GUIDO GERKEN, DONGLIANG YANG, JOERG F. SCHLAAK

**Affiliations:** 1 Department of Gastroenterology and Hepatology, University Hospital of Essen, Essen D-45122, Germany; 2 Department of Infectious Disease, Union Hospital, Tongji Medical College, Huazhong University of Science and Technology, Wuhan, Hubei 430022, P.R. China; 3 Department of Ultrasonography, Union Hospital, Tongji Medical College, Huazhong University of Science and Technology, Wuhan, Hubei 430022, P.R. China; 4 Institutes of Diagnostic and Interventional Radiology and Neuroradiology; 5 Nuclear Medicine, University Hospital of Essen, Essen D-45122, Germany

**Keywords:** hepatocellular carcinoma, transarterial radioembolization, tumor response, Choi criteria, RECIST, mRECIST

## Abstract

In this study, Response Evaluation Criteria in Solid Tumors (RECIST), modified RECIST (mRECIST), Choi and modified Choi criteria were compared to determine which method is optimal for response evaluation in hepatocellular carcinoma (HCC) patients treated with transarterial radioembolization (TARE) with yttrium-90 microspheres. Responses were evaluated by RECIST, mRECIST, Choi and modified Choi criteria in 113 patients with HCC undergoing TARE. Results were compared at 12 weeks after therapy. Kaplan-Meier survival analyses and Cox regression were used to assess differences in time to progression (TTP) and overall survival (OS) between the responders and non-responders defined by each method. The results demonstrated that the responders and non-responders defined by mRECIST and Choi criteria successfully identified patients with a long TTP (400 and 280 days) or short TTP (188 and 166 days) (P=0.004 and 0.002, respectively). Neither RECIST nor modified Choi criteria discriminated between patients who had a short or long clinical benefit. Cox regression analysis revealed that Choi response was a prognostic factor of OS (P=0.004) and was associated with a 53% risk reduction. There was no significant association between survival and RECIST, mRECIST and modified Choi responses. In conclusion, tumor response according to Choi criteria may be helpful to define early HCC patients who benefit from TARE. RECIST, mRECIST and modified Choi appeared inferior.

## Introduction

Hepatocellular carcinoma (HCC) is the most common primary malignancy of the liver. The prognosis of patients with HCC is generally poor, as in the majority of cases, HCC is diagnosed at an intermediate or advanced stage (1), and curative treatments (resection, transplantation and radiofrequency) are only suitable for early stage HCC patients (2). Transarterial radioembolization (TARE) with yttrium-90 microspheres is an emerging tool for the treatment of primary and metastatic HCC (3). It produces average disease control rates >80% and is usually extremely well-tolerated (4).

Tumor response has been considered to be pivotal for surrogate assessment of therapy efficacy. Response Evaluation Criteria in Solid Tumors (RECIST) has been the standard method for evaluation of solid tumors since its introduction in 2000 (5). A RECIST-defined response depends on the change in size of target lesions, determined by non-invasive imaging assessment, while a revised guideline known as the modified RECIST criteria (mRECIST) takes into consideration changes in the degree of tumor arterial enhancement (6). TARE may lead to disease stabilization without actual shrinkage of tumor size, but with a decrease in hypervascularity and the presentation of necrosis. Therefore, evaluation based on tumor size alone, as RECIST or mRECIST, may no longer be adequate for modern tumor therapy follow-up. Choi *et al* have developed new criteria for gastrointestinal stromal tumors (GIST), which assess a change in size or a change in density of target lesions. Choi criteria appear to be better predictors of clinical response to imatinib than RECIST (7). However, these criteria have not been extensively evaluated in HCC patients treated with TARE.

In this study, we compared tumor responses according to RECIST, mRECIST, Choi and modified Choi criteria in HCC patients treated with TARE, and investigated their association with time to progression (TTP) and overall survival (OS).

## Patients and methods

### Patients

The records of patients who were treated with yttrium-90 TARE for intermediate or advanced HCC at the University Hospital of Essen (Essen, Germany) from June, 2008 until December, 2012 were reviewed. A total of 149 patients were identified, of which 36 were excluded due to either incomplete imaging or a follow-up period of <12 weeks. All data were analyzed retrospectively in an anonymous fashion according to the principles expressed in the Declaration of Helsinki. This study was approved by the institutional review boards of the University Hospital of Essen (Essen, Germany).

### Treatment

The microspheres used were glass-based (TheraSphere, Ottawa, ON, Canada) and were composed of 20–25 μm particles. Pretreatment mesenteric angiography and technetium-99m macroaggregated albumin scans were performed to assess gastrointestinal flow and lung shunting (8). For evaluation of TARE efficacy, 12 weeks after the initial treatment a physical examination, abdominal computed tomography (CT) scan and blood tests were performed. Thereafter, assessment was performed every 12 weeks.

### Radiological assessment of response

Assessment was performed by contrast-enhanced spiral CT. Treatment responses were evaluated by RECIST, mRECIST, Choi and modified Choi criteria, in which a response is based on both a minimum of a 10% reduction in size and a 15% reduction in density (9,10). The four imaging criteria are shown in Table I. All criteria encompassed the following four response categories: Complete response (CR), partial response (PR), stable disease (SD) and progressive disease (PD). The CT attenuation of each tumor was measured in Hounsfield units (HUs) on the portal venous phase. The HU density value was obtained by delineating a region of interest (ROI) around the boundary of the entire tumor at baseline and 12 weeks after TARE. The HUs of all lesions were combined and a mean for each patient was calculated as described previously (7).

For calculation of the TTP, radiological progression defined by these four methods was used. TTP was defined as the number of days from the start of therapy to the day in which progression was confirmed. Mortality during follow-up without evidence of radiological progression was censored. Survival time was evaluated as the time between initial treatment and mortality or loss to follow-up (11). For TTP and OS analyses, data collection was terminated on December 31, 2012.

### Statistical analysis

Statistical analysis was performed using SPSS 18.0 software (SPSS Inc., Chicago, IL, USA). For the analyses according to the four different criteria, patients were categorized into responders (CR + PR) vs. non-responders (SD + PD). Kaplan-Meier survival analyses and Cox regression were used to explore differences in TTP and OS between the responders and non-responders according to RECIST, mRECIST, Choi and modified Choi criteria. Spearman’s correlation test was performed to detect possible correlations. The Wilcoxon signed-rank test was used to compare the changes in size and attenuation at baseline and 12 weeks after TARE. P<0.05 was considered to indicate a statistically significant difference.

## Results

### Baseline demographics

A total of 113 HCC patients treated with TARE were enrolled in this study. Table I summarizes the baseline demographics of the cohort. The median age was 69 years (range, 19–88 years). The majority of the patients were male (80%) and the predominant etiology of liver disease in this European cohort was non-alcoholic steatohepatitis. The median model for end-stage liver disease score was 8 (range, 6–26). Approximately half the patients (48%) had radiological evidence of cirrhosis.

### Response according to RECIST and mRECIST

For RECIST response, at 12 weeks post-TARE, all lesions presented a median change in tumor size of −15% (range, −100 to +31%). Twenty-five (22%) patients reached PR, 75 (66%) patients had SD and 13 (12%) patients had PD, resulting in 25 responders and 88 non-responders. For mRECIST response, the median change in tumor size was −16% (range, −100 to +119%). Thirty (27%) patients reached PR, 61 (54%) patients had SD and 22 (19%) patients had PD, hence 30 responders and 83 non-responders.

### Response according to Choi and modified Choi criteria

At baseline, the median tumor size was 77 mm (range, 15–229 mm) for all lesions, with a median attenuation of 37 HUs (range, 12–80 HUs). At 12 weeks post-TARE, the median size and attenuation had decreased to 56 mm (range, 0–199 mm; P<0.05) and 29 HUs (range, 10–70 HUs; P<0.05), respectively. Overall, there was a weak correlation between the percentage change in tumor size and the percentage change in attenuation (Spearman’s ϱ=0.161, P=0.044).

On evaluation with Choi criteria at 12 weeks post-TARE, 88 (78%) patients reached PR, 20 (18%) had SD and 5 (4%) had PD, resulting in 88 responders and 25 non-responders. According to modified Choi criteria, 40 (35%) patients reached PR, 68 (60%) had SD, and 5 (4%) had PD, hence 40 responders and 73 non-responders.

### Association with TTP and OS

**Figure 1:**
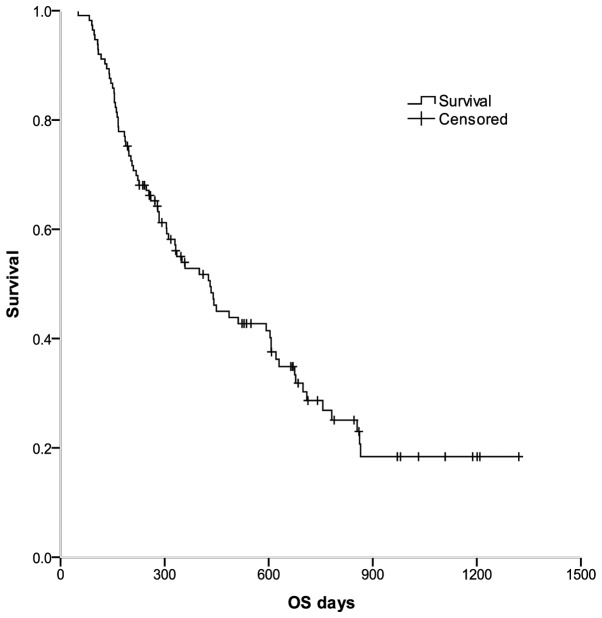
OS in the whole series. Kaplan-Meier curves were generated to show the OS of all HCC patients following TARE. OS, overall survival; HCC, hepatocellular carcinoma; TARE, transarterial radioembolization.

As shown in Fig. 1, for all 113 patients, the median OS was 431 days [95% confidence interval (CI), 321–541 days], and the 1- and 2-year survival rates were 52.8 and 28.7%, respectively.

**Figure 2:**
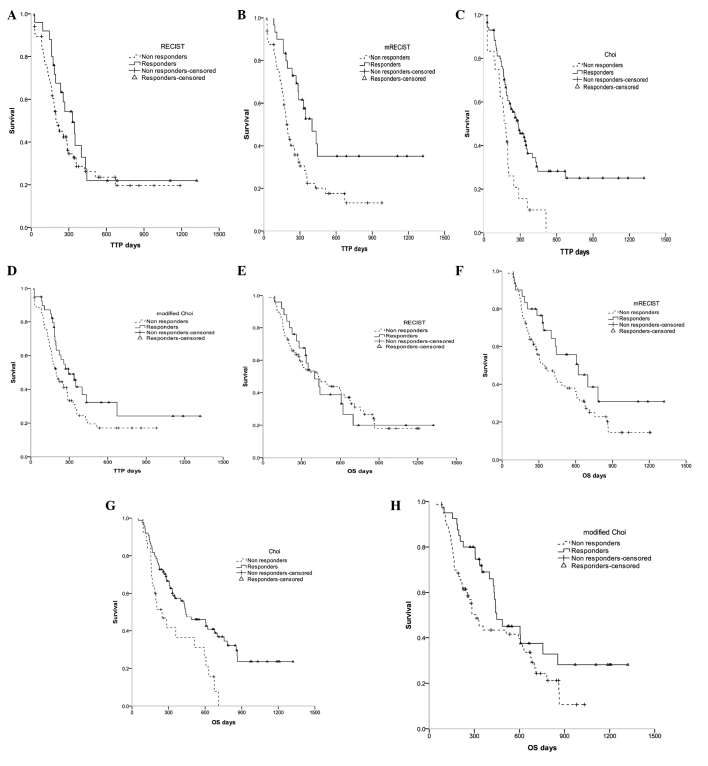
Kaplan-Meier curves were generated to compare TTP and OS between responders and non-responders according to four radiological assessment methods. HCC patients undergoing TARE had radiological responses, as evaluated by four criteria: (A and E) RECIST, (B and F) mRECIST, (C and G) Choi and (D and H) modified Choi, performed 12 weeks post-TARE. TTP and OS were compared between responders and non-responders, according to the different criteria. TTP, time to progression; OS, overall survival; HCC, hepatocellular carcinoma; TARE, transarterial radioembolization; RECIST, Response Evaluation Criteria in Solid Tumors; mRECIST, modified Response Evaluation Criteria in Solid Tumors.

Kaplan-Meier methods were used to calculate the median TTP and OS for the responders and non-responders defined by each of the four criteria (Fig. 2, Table III). The results demonstrated that patients who had a response according to Choi criteria had a significantly longer TTP and OS compared with those who were non-responders (median, 280 vs. 166 days and 442 vs. 247 days; P=0.002 and 0.003, respectively; Fig. 2, Table III). The responder and non-responder groups defined by mRECIST also demonstrated a significant difference in median TTP (P=0.004; Fig. 2, Table III). Neither RECIST- nor modified Choi criteria-defined responders exhibited a significant improvement in TTP and OS compared with the non-responders (Fig. 2, Table III).

Cox regression based on either RECIST, mRECIST, Choi or modified Choi criteria as covariates was generated to compare TTP and OS between responders and non-responders, according to the four response criteria (Table IV). When mRECIST criteria was used, hazard ratios (HRs) for TTP and OS in responders compared with non-responders were 0.42 (95% CI, 0.24–0.74) and 0.61 (95% CI, 0.35–1.06), respectively (P=0.003 and 0.080, respectively; Table IV). According to the Choi criteria, the differences in HRs for TTP and OS between responders and non-responders were significant [0.46 (95 CI, 0.28–0.77) and 0.47 (95% CI, 0.28–0.78); P=0.003 and 0.004, respectively; Table IV]. Choi responders had a 53% risk reduction for OS compared with that of non-responders.

## Discussion

HCC is usually a hypervascular tumor (12) and CT scanning has improved our ability to detect HCC by allowing acquisition of hepatic arterial and portal venous dominant sets of images (13). TARE with yttrium-90 microspheres is an established local-ablative therapy for primary and metastatic liver cancer, and it has shown promising efficiency (3,4). Imaging-defined response assessment based solely on change in tumor size may be appropriate for treatments that result in significant tumor shrinkage; however, TARE may cause tissue necrosis with no immediate change in tumor size. Hence the mRECIST, Choi and modified Choi criteria, which measure hypervascular tumors, have been proposed as alternatives to RECIST. The Choi criteria define a partial response by either a 10% reduction in size or a 15% reduction in density during the portal venous phase of contrast. It has been suggested that the Choi criteria may be appropriate for tumor response assessment in GIST cancer (7). However, these four methods have not been directly compared, nor has their association with survival, when measured at a single time-point in a series of patients treated with TARE alone.

In this retrospective study, we focused on the use of TARE for HCC patients and compared the RECIST, mRECIST, Choi and modified Choi criteria. The OS and TTP are the major endpoints for clinical trials in HCC (11). We evaluated the ability of each criteria-defined response to correlate with TTP and OS in HCC patients treated with TARE. Our results demonstrated that for CT assessments, at the 12-week follow-up of 113 HCC patients, neither RECIST- nor modified Choi criteria-defined responses correlated with TTP. However mRECIST and Choi criteria successfully identified that responders had an extended TTP, while non-responders had a significantly shorter TTP. Furthermore, the OS between the responders and non-responders according to RECIST, mRECIST and modified Choi criteria were not significantly different at the 12-week follow-up. Patients who had a response according to Choi criteria had a significantly longer OS compared with the non-responders.

We also investigated which response assessment has an association with overall survival. Previous studies have shown that World Health Organization, RECIST and European Society for the Study of the Liver responses are associated with improved survival (14,15). Our findings clearly revealed that overall response according to Choi criteria was a prognostic factor of survival and associated with a 53% reduction in risk of mortality. There was no significant association between overal survival and RECIST, mRECIST and modified Choi responses in this study. Choi criteria had a significantly better predictive value compared with the other criteria for TTP and OS in HCC patients treated with TARE at 12-weeks follow-up and may be valuable for making early decisions on whether current interventions should be continued or altered.

However, there are several limitations in the use of the Choi criteria for evaluation of TARE-induced responses in HCC. Firstly, the best method to evaluate tumor density using ROI analysis remains a topic of debate (16). Secondly, measurements of relatively hypodense lesions at baseline may be less reliable, since a 15% decrease in HUs of these lesions is less accurate than those in lesions with higher pretreatment HUs. Therefore, the use of absolute changes may be more appropriate than the percentage change in HUs (17). Thirdly, compared with RECIST, Choi criteria are not able to identify patients with PD early, possibly due to the ≥10% increase in tumor size compared with the ≥20% increase used by RECIST.

In conclusion, tumor response assessment according to Choi criteria at the 12-week follow-up in HCC patients treated with TARE distinguishes prognostic groups better than RECIST, mRECIST and modified Choi criteria. This may allow early discrimination of patients benefiting from further treatment and those who will not, particularly patients with stable tumor size. This small sample study requires validation by further, larger prospective treatment studies to demonstrate the broader applicability of the Choi criteria.

## Figures and Tables

**Table I:** Definition of target radiological responses.

Response	RECIST 1.1	mRECIST	Choi criteria	Modified Choi criteria
CR	Disappearance of all target lesions	Disappearance of any intratumoral arterial enhancement in all target lesions	Disappearance of all target lesions	Disappearance of all target lesions
PR	At least a 30% decrease in the sum of the greatest unidimensional diameters of target lesions	At least a 30% decrease in the sum of unidimensional diameters of viable target lesions	Decrease in tumor size ≥10% or decrease in tumor density ≥15% on CT	Decrease in tumor size ≥10% and decrease in tumor density ≥15% on CT
SD	Any cases that do not qualify for either partial response or progressive disease	Any cases that do not qualify for either partial response or progressive disease	Does not meet the criteria for CR, PR or PD	Does not meet the criteria for CR, PR or PD
PD	An increase of at least 20% in the sum of the diameters of target lesions	An increase of at least 20% in the sum of the diameters of viable target lesions	Increase in tumor size ≥10% and does not meet PR criteria by tumor density	Increase in tumor size ≥10% and does not meet PR criteria by tumor density

CR, complete response; PR, partial response; SD, stable disease; PD, progressive disease; CT, computed tomography.

**Table II:** Patient baseline demographics and tumor characteristics.

Variable	Value
Age, years	69 [19–88]
Male	90 (80)
Etiology of HCC	
HCV	18 (16)
HBV	17 (15)
HBV + HCV	8 (7)
NASH	35 (31)
Other	12 (11)
Cryptogenic	23 (20)
MELD score	8 [6–26]
Bilirubin, mg/dl	0.7 [0.2–4.9]
INR	1.05 [0.88–2.76]
Creatinine, mg/dl	0.99 [0.66–8.00]
ALT, U/l	44 [13–285]
AST, U/l	53 [17–299]
Cirrhosis	54 (48)
AFP, U/ml	54.0 [0.8–55791.0]
Number of lesions	
1	56 (50)
2–5	35 (31)
>5	22 (19)
Size of the largest lesion, cm	
≤3	16 (14)
3–5	33 (29)
5–10	45 (40)
>10	19 (17)

Data are presented as N (%) or median [interquartile range]. HBV, hepatitis B virus; HCV, hepatitis C virus; NASH, non-alcoholic steatohepatitis; MELD, model for end-stage liver disease; INR, international normalized ratio; ALT, alanine transaminase; AST, aspartate transaminase; AFP, α-fetoprotein.

**Table III:** Responses according to RECIST, mRECIST, Choi and modified Choi criteria and association with TTP and OS.

	TTP^a^			OS^a^		
		
Response criteria	Responders	Non-responders	P-value	Responders	Non-responders	P-value
RECIST	330 (207–453)	203 (146–260)	0.270	400 (259–541)	431 (241–621)	0.965
mRECIST	400 (273–527)	188 (164–212)	0.004	621 (278–964)	332 (192–472)	0.077
Choi	280 (191–369)	166 (129–203)	0.002	442 (250–634)	247 (123–371)	0.003
Modified Choi	294 (179–409)	197 (148–246)	0.072	449 (271–627)	311 (231–391)	0.069

a Median number of days with 95% CIs.

RECIST, Response Evaluation Criteria in Solid Tumors; mRECIST, modified Response Evaluation Criteria in Solid Tumors; TTP, time to progression; OS, overall survival.

**Table IV:** Cox regression was generated to compare TTP and OS between responders and non-responders according to the four response criteria.

		TTP		OS	
			
Response criteria	n	HR (95% CI)	P-value	HR (95% CI)	P-value
RECIST					
Responders	25	0.74 (0.43–1.27)	0.274	0.99 (0.58–1.70)	0.965
Non-responders	88	1.0		1.0	
mRECIST					
Responders	29	0.42 (0.24–0.74)	0.003	0.61 (0.35–1.06)	0.080
Non-responders	84	1.0		1.0	
Choi					
Responders	88	0.46 (0.28–0.77)	0.003	0.47 (0.28–0.78)	0.004
Non-responders	25	1.0		1.0	
Modified Choi					
Responders	40	0.64 (0.40–1.05)	0.075	0.64 (0.39–1.04)	0.072
Non-responders	73	1.0		1.0	

RECIST, Response Evaluation Criteria in Solid Tumors; mRECIST, modified Response Evaluation Criteria in Solid Tumors; TTP, time to progression; OS, overall survival; HR, hazard ratio; CI, confidence interval.
